# Diversity, friction, and harmonisation: an ethnographic study of interprofessional teamwork dynamics

**DOI:** 10.1186/s12913-022-07596-0

**Published:** 2022-02-19

**Authors:** Henriette Lund Skyberg

**Affiliations:** Department of Social Work, Child Welfare and Social Policy, Faculty of Social Sciences, Oslo Metropolitan University, St. Olavs plass, P.O. Box 4, NO-0130 Oslo, Norway

**Keywords:** Interprofessionalism, Teamwork, Team dynamics, Health services, Social work, Ideal-type model, Mental Health, Substance use, Norway

## Abstract

**Background:**

Although diversity, friction, and harmonisation in interprofessional teamwork are aspects frequently conceptualised, no empirical study discusses them in combination. Focusing on risk and function with respect to each aspect, this article empirically examines how dynamics between these aspects during interprofessional teamwork interactions fosters conditions for effective teamwork.

**Methods:**

An ethnographic study of three interprofessional teams, in the context of mental health and substance use, was conducted in Norway. Data were collected through observations of 14 team meetings and 18 in-depth interviews with health and social work professionals. Thematic analysis was applied to code the data.

**Results:**

A conceptual ideal-type model, which includes all three aspects was developed to represent the emergent findings. The results suggest that the diversity of professional perspectives inherent in interprofessional teams is the foundation of interprofessional teamwork. However, friction is needed to promote innovation, encourage new insights, and intensify discussions. In addition, harmonisation balances professional distinctions, fosters trust, and ties professionals together.

**Conclusion:**

This article presents a comprehensive model of how professionals work together in interprofessional teams. The model makes visible the functions and risks of each aspect and the dynamics between them. Furthermore, the article argues for mobilisation and balance of all three aspects in combination to maximise the capacity of interprofessional teamwork. Such insight can be used to support the development and successful implementation of interprofessional teamwork in health care.

## Background

Interprofessional teamwork can be conceptualised as active and ongoing partnership between professionals with diverse backgrounds and distinctive professional cultures working together to solve problems and provide services [1]. However, although interprofessional teams focus on collaboration, on the sharing of knowledge and complementary skills, they also face several challenges, including conflicting perspectives, poor communication, role conflicts, and confusion [2]. In addition to barriers at the team level, studies on interprofessional teamwork report on issues at an organisational level as well, such as differences in professional culture, power, and policy [3].

This article reports on the results of an ethnographic study of three interprofessional teams in the field of mental health and substance use in Norway. The starting point for the study was to explore how social work and health care professionals worked together as a team. When working with these data, it became apparent that informants highlighted diversity in professional perspectives and the friction between such perspectives as effective, while at the same time emphasising elements of harmonisation as critical to group functioning. To explore this further, a search for literature discussing these topics was conducted. Much of the literature reviewed focused on three corresponding aspects of interprofessional teamwork 1) diversity, the inclusion of different types of professionals (inherent in interprofessional teams); 2) friction, the intersection of ideas among professionals with divergent interests and resources; and 3) harmonisation*,* which involves elements that can bind diverse, distinctive professionals and potentially opposing forces together. However, although all three aspects were well accounted for in the literature, no empirical studies discussing all three aspects in combination were found.

Since the three aspects are often referred to, but no synthesis or relational analysis of them have been made, it seems fruitful to study the connection between them more closely. In this, lies a critique of the literature that has dealt with each aspect separately. Studying the three in combination is important as it highlights each aspect’s function and risks associated with it. Furthermore, examining the dynamics between them helps to understand ‘effective work’ in the context of interprofessional teamwork and how to facilitate it in practice.

Based on the hypothesis that all three aspects have functions and involve risks in interprofessional teamwork, in the sense that each aspect does something to the teamwork, this article examines how the dynamics between these aspects can create conditions for effective teamwork. Here, ‘effectiveness’ refers to a comprehensive combination of professional competence and skills and is related to the achievement of goals, team performance, and what professionals can accomplish together [3]. It denotes a dynamic where something other than the simple sum of each team member’s individual competence is created. For example, although friction between diverse professional perspectives can be problematic for team functioning as opposing opinions may lead to conflict, it can also have positive effects on team performance by evoking innovation and creativity [3, 4].

The importance of interprofessional teamwork in the present context stems from the complex and multifaceted nature of patients’ health and social care. For example, in the field of mental health and substance use, patients’ problems are often notably compound and intertwined with other health and social problems. The achievement of comprehensive care and treatment for these patients requires close interaction and collaboration between different professionals with various, and sometimes, conflicting perspectives. Although this article is related to a specific national context and field of practice, the results are likely relevant to other contexts as there is increasing international support for interprofessional work in health and social services.

By studying all three aspects in combination through ethnographic data, this article provides an empirical and analytical model for understanding how professionals work together as a team. The focus of the model is on the functions of each aspect, the risks involved, and dynamics between the three aspects. That being said, in line with the methodological tradition of Weber [5], the interconnection of the three aspects in this model should be understood as an ‘ideal type’, meaning that it presents typical characteristics and elements found in interprofessional teamwork, rooted in empirical studies of human action and motives. An ideal type, such as this one, is a useful tool for focusing on specific aspects of interprofessionalism, but it does not fully reflect all aspects of interprofessional teams. The reality is always far more complex, and by adding a different perspective (power, for example), other characteristics could emerge as more prominent.

### Literature review

As a field of research, interprofessionalism is fragmented and characterised by several analytical and theoretical perspectives such as professional boundaries and power, as well as processual factors such as time, space, and the interactional determinants of interprofessional collaboration [3, 6, 7]. In this article, the analytical perspective is ‘team dynamics’, defined as the interaction between individuals with complementary skills who are committed to a common working approach, purpose, and performance toward goals, for which they hold each other mutually responsible [8]. In exploring the literature on such dynamics, the three above-noted aspects were found to be particularly prominent. Although not necessarily using the terms harmonisation, diversity, and friction themselves, several studies have discussed what the terms imply. The following review is based on the author's interpretation and synthesis of the literature.

Starting with the first aspect, harmonisation*,* it can be understood as elements that minimises conflict and create a balanced composition of individual parts. In much literature, factors such as respect, trust, and the mutual acknowledgement of professional roles have been highlighted as conflict-reducing factors [9], which makes it possible to interpret these as studies of harmonising. Karam et al. [10], for example, argued for mutual respect as a key element in balancing power among professional groups. In comparison, Pullon [11] found that respect and understanding of each other’s professional roles led to interprofessional trust which in turn helped in resolving potential conflicts in constructive ways. Similarly, according to McDonald et al. [12], respectful interactions based on trust promoted interprofessional collaboration. Lastly, Flood et al. [13], found that interprofessional work flourished when professionals were open and responsive to the situation and each other.

The studies above point to elements that bind professionals together, creating connections between distinct and potentially conflicting forces. In more recent literature, these connections have been linked to the theoretical concept of ‘team psychological safety’, meaning a shared belief within a team that it is safe for interpersonal risk taking [14, 15]. However, as each health and social work profession has its own culture: values, beliefs, attitudes, customs, and behaviours [16], professionals from different disciplines may look at the same situation and see different features [17]. Accordingly, other studies have shown how the absence of harmonising elements can present barriers to interprofessional work. Reeves et al. [18] and Atwal and Caldwell [19], for example, both argued that professional hierarchies and status imbalances hindered effective interprofessional work by preventing lower-status professionals from contributing with their perspectives. Simultaneously, too much similarity entails a risk of over-harmonisation, which in turn can reduce opportunities for creative tension or the ability to challenge norms within a team [20].

Moving to the second aspect, diversity refers to the existence of autonomous professionals with different knowledge and skills within an interprofessional team and is an inherent quality of such teams. Such diversity can help in increasing the collective competence of the team and provide access to a wider experience that can inform the team. In several studies, researchers have argued that the experience of participating in interprofessional work can encourage the development of boundary setting and, as a result, a clearer diversity in professional roles. In studying community mental health teams, B. Brown et al. [21] found that drawing boundaries was a strategy professionals could use to set limits, taking ownership, and encourage responsibility for tasks. Similarly, MacNaughton et al. [22] argued that professional autonomy was an important element of interprofessional team functioning and revolved around interprofessional interactions and the distribution of tasks. Thylefors [23] discussed how different problems demanded different prerequisite knowledge and pointed towards the need for ‘functional influence’, meaning that the members most competent to speak on an issue should say the most.

Despite its value, the diversity inherent in interprofessional teams can lead to problems. Interprofessional teams diverge from other types of organisations as team members may have allegiances not just to the team but also their own profession. Here, Rose [24] found that in the process of reaching an agreement, professional diversity risked leading to negotiation and professional self-sacrifice. In comparison, Lewin and Reeves [25] argued that, within a hospital context, the differences between doctors and nurses resulted in relationships characterised by limited information sharing, which in turn risked leading to parallel discussions and series of discussions with limited intersection.

Looking at the third aspect, friction*,* it has been argued that an absence of disagreement and debate within a team can threaten a team’s creativity and innovation [3, 4, 26]. In the context of interprofessional teamwork, friction refers to the intersection of ideas between professionals with divergent interests, resources, and status. The friction aspect comprises both risks and functions, by being either an ‘effective force’ or a ‘source for conflict’. While friction as an effective force refers to differences that lead to reflection and improvement, friction as conflict refers to a disruptive clash of interest. Studying multiple interprofessional health care teams, J. Brown et al. [27] for example, identified professional power hierarchies and the devaluation or degradation of the perspectives of lower status professionals both as sources to conflict and as a barrier to conflict resolution. According to J. Brown et al. friction could, for example, become a conflict if there was a lack of recognition or motivation to address disagreement. On the contrary, other studies have argued that an absence of friction within a team can lead to over-harmonisation or ‘groupthink’, that is, lack of disagreement and debate between team members [3]. In discussing the merits of interprofessional collaboration, Kaba et al. [28] warned that symptoms of groupthink include collective rationalisation, self-censorship, direct pressure on dissenters, and self-appointed ‘mind guards’ that can lead to poor decision making. Following McMurtry et al. [29], professionals with the same background may share biases; shared biases can result in a failure to question assumptions and to avoidance of uncomfortable debate. Conversely, friction between diverse perspectives minimises the danger of the ‘groupthink’, and instead includes a potential ‘learning zone’ [30].

All in all, what the literature suggests is that harmonisation implies the presence of conflict-reducing factors that can help professionals orient towards common goals and that diversity and friction are essential to effectiveness (in the distribution of tasks and responsibilities) and team creativity. However, although some studies have discussed how professionals work together to overcome barriers of professional diversity [1, 31], or how team collectivism has an indirect impact on interprofessional success [32, 33], no known study has empirically discussed all three aspects in combination. Since the three aspects are clearly central to teamwork and also seem to be interconnected, it is important consider these aspects in connection in order to understand the dynamics in interprofessional teamwork. This approach addresses a gap in the research field of interprofessionalism.

### The need for a threefold model

**Fig. 1:**
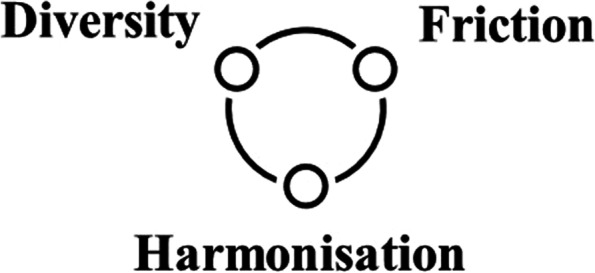
Threefold model of interprofessional team dynamics

By studying interactions between diversity, friction, and harmonisation more closely, this article argues that links between these aspects represent an essential dynamic in interprofessionalism and influences teamwork effectiveness. Having considered the empirical data altogether, a threefold model conceptualising the aspects according to function, risk and dynamic was constructed. These three factors were chosen as they help to shed light on the conditions for effective teamwork. For example, the function of diversity is to give access to different professional knowledge and perspectives. When different professional perspectives intersect, friction arises which also creates conditions for creativity. However, both diversity and friction involve risks. Too much friction without group harmonisation may, for example, lead to conflict. In other words, by discussing the three aspects in combination, this article illustrates how each aspect represents an effect on teamwork both in itself and in a dynamic interaction with the other aspects (Fig. 1). This model will be further elaborated, when discussing the findings.

Studying the three aspects in combination can lead to further development of analytical concepts and dynamics related to interprofessionalism. The presented aspects represent ideal features that can be found in interprofessional teamwork, and for each aspect, certain outcomes can be predicted. This ‘ideal-typical model’ invites a more comprehensive, meaningful discussion on the processes of interprofessionalism teamwork than those found in literature that treats them separately. For example, the model reveals how harmonisation can affect the functions of friction and diversity and their associated risks. It should be noted that the three aspects are not the result of causal mechanisms, but something that is created through the team members’ motives and intended actions. Furthermore, these are not the only aspects of interprofessional teamwork; professional power, organisational framework and jurisdictional boundaries are also much discussed in the literature [34, 35]. However, when analysing the data, these three aspects were judged by the author to be prominent and clearly empirically connected.

## Methods

### Research setting

The three teams studied were selected based on two criteria: 1) they included both social work and health care professionals, and 2) were organised according to an interprofessional team model—implying high levels of communication, mutual planning, collective decisions, and shared responsibilities between the professionals [36].

All three teams served densely populated urban areas and included 8–14 professionals with backgrounds in nursing, occupational therapy, psychiatry, clinical psychology, social education (this is a bachelor’s degree in Norway), and social work. The patient groups for all teams included people over the age of 18 with mental health- or substance use-related issues or both. One team worked at a daytime clinic that provided only short-term follow-up with patients whose challenges were less extensive than those of the other two teams. The other two teams worked in outreach and provided extended follow-up care (Table 1).

**Table 1:** Summary of the interprofessional teams

	Number of members	Composition of professions	Structure
Team 1	8	Psychology, social work, nursing, and medicine	- Daytime clinic - Short-term treatment - Patients’ challenges less extensive - Team meetings once a week
Team 2	10	Social work, social education, psychology, and medicine	- Outreach and extended follow-up care - Long-term treatment - Patients’ challenges extensive - Team meeting once a week
Team 3	14	Nursing, medicine, psychology, social work, social education, and occupational therapy	- Outreach and extended follow-up care - Long-term treatment - Patients’ challenges extensive - Team meetings every morning

In this study, all members of each team had a list of patients they were responsible for, either individually or with a colleague. The goals of all three teams were treatment, rehabilitation, and social support for patients. Tasks performed by the teams included medical and mental assessments, providing treatment for mental illness and substance use, and assisting patients with their finances, housing, education, work, and social life. In all teams, each professional’s role was autonomous, although all members worked closely together.

In team meetings, members distributed tasks and discussed patient cases. The main purpose of the meetings was to share information about patients (status and plan for treatment) and receive input from colleagues about complex problems. For example, how can we help the client to find a more permanent housing? Consequently, meetings functioned more as forums for discussion than for decision-making. In addition to team meetings, the professionals had daily contact with one another through consultation and collaboration with patients.

### Data collection

Data were collected between April and December 2019 and comprised 14 observation sessions and 18 in-depth interviews (Table 2). The teams were recruited through the professional network of the author who previously held a position in the administration of a mental health service, though not as a health or social worker but as a social scientist. The author had no direct affiliation with the teams prior to the study.

**Table 2:** Data collection

	Number of team members	Number of team members interviewed	Number of observation sessions
Team 1	8	4	7 (14 hours)
Team 2	10	7	7 (21 hours)
Team 3	14	7	0
Total	32	18	14 (35 hours)

Observations were collected during participation in weekly team meetings lasting two to three hours. The focus of the observations was ‘how the professionals discussed patient cases’, including types of questions asked, who asked them, and answers given. Fieldnotes including keywords and near-verbatim quotes were hand-written during the observation sessions and then typed into a Word document in more detail after the session on the same day. All notes were first written in Norwegian and later translated into English by the author.

Due to patient confidentiality issues, the sessions were not recorded, and ethical approval for observation was obtained for two of the three teams only; anonymising patient data with the third team would not have been possible. Only interviews were conducted with the third team. If this represents an imbalance in the data, it is assumed to have had little effect on the findings as the purpose of having three teams was to collect richer and more extensive data, and not conduct a comparative study.

Team members were contacted with requests for interviews, and those who consented were interviewed. To secure informant anonymity, the number of workers interviewed from each profession will not be disclosed; however, one to three representatives from each profession were interviewed. Informants were questioned about their professional roles and experiences with interprofessional work. The semi-structured interviews with informants were guided by open-ended questions asking them to reflect on the topics at hand; for example, ‘Have you experienced other professionals having a different perspective than you?’, was followed-up with ‘In which situations?’, and ‘How was this handled?’ The interviews were conducted after the observations. Consequently, several of the questions were based on observational data, for example, questions about specific events that had occurred in those sessions. The interviews were recorded and later transcribed and translated into English by the author.

All participants were informed about the project both orally and in writing and gave written consent to participation. The study was approved by the Norwegian Regional Committees for Medical and Health Research Ethics (approval reference 2019/809) and the Norwegian Centre for Research Data (approval reference 237074).

### Data analyses

Inspired by Braun’s and Clarke’s [37] approach to thematic analysis, the author coded the data in two rounds using NVivo12 software. The first round was open-ended, aimed at forming an overview of the data and revealing notable dynamics between professional autonomy and team collectivism. After the first round of coding, the three mentioned aspects of interprofessional teamwork were found to be relevant. However, as previously mentioned, although all three aspects were found in the literature, there are no studies that discuss them in combination. Therefore, a second round of coding was conducted, organising the data in the following themes:

1. Diversity: an inherent quality of a team of autonomous professionals with different knowledge and skills.
2. Friction: intersections of ideas between professionals with divergent perspectives and resources.
3. Harmonisation: comprises elements that can bind diverse, distinctive professionals and potentially opposing forces together.

The definition of each aspect came out of a combination of how previous literature had described similar features and the empirical data in this study. When analysing the data, how these aspects manifested the function, risk, and dynamic between them were noted. Here, the interviews provided the professionals’ subjective reflections, and the researcher’s observations described the behaviour and context in which they arose. Regarding the use of the term effective, no separate objective or quantitative assessment was made of success and failure. The idea of effectiveness was based on what the informants expressed in interviews.

## Results and Discussion

### Diversity

When discussing patients’ cases, the professionals typically presented differing perspectives. This was manifested in at least two ways. First, as some form of ‘functional influence’ [23] and the idea that the members most competent to speak on an issue were expected to contribute the most [22, 23]. Second, the data suggests that in these diverse groups there is a risk of professional perspectives and roles co-existing without intersecting, resulting in parallel or series of perspectives [25]. The two following empirical examples illustrate both. The first is an observation from a team meeting:

The team was discussing a case where they were unsure of the exact nature of patient’s problems. A social educator, who had the main responsibility for following up with the patient, started the discussion.

Social educator: ‘I had a meeting with [the patient] yesterday. I asked if [the patient] was willing to agree to hospital admission for a mental assessment. [The patient] does not want that. [The patient] only wants to change the medication. (…) I think [the patient] is in a desperate situation with a lot of debt and a lot of anxiety, and the medicine only makes [the patient] worse.’

One psychologist responded: ‘It becomes difficult when we are unsuccessful in making a proper mental assessment (…) The medicine [the patient] is taking is probably not right. [The psychiatrist] should have a look at it [the psychiatrist was not present at the meeting].

The team continued discussing other possible strategies for helping the patient.

Social worker #1: ‘When it comes to [the patient’s] finances, it’s under the administration of social services.’

Social educator: ‘There are a lot of things we do not know. Does [the patient] have problems with substance use?’

Social worker #1: ‘I don’t know. I’ve never observed [the patient] intoxicated.’

Social worker #2: ‘What if we get [the patient] into “supported housing”? Then we can get closer and observe [the patient].’

Although the discussion ended without members determining concrete solutions or making decisions, the function of diversity is illustrated in the multiple professional perspectives that were presented. While the psychologist addressed the need for a mental assessment and (by deferring discussion to the psychiatrist) change of medication, the social workers addressed issues such as substance use, finances, and housing. Consequently, different parts of the patient's possibly compound problem were considered by professionals in the relevant fields, leading to a sense of ownership in tasks for each. Furthermore, a form of functional influence can be observed considering that with the absence of the psychiatrist, questions of changes to the patient’s medication fell out of the discussion and the social work perspectives were more prominent.

The concept of functional influence helps shed light on how the process of solving problems promotes professional diversity in terms of professional roles and its associated expectation. In interviewing a team leader about team discussions of new patient cases, such divisions between professionals became even clearer:

We often start by discussing the patient’s need for treatment and follow-up care. Does the patient have any mental health issues? If so, it becomes natural to involve the psychologist. Does the patient have any somatic diseases? Maybe the psychiatrist should have a look at the patient. Substance use? Problems related to housing and economy? Does the patient need other services [outside the team]? Social services, for example. For such topics, the social worker often makes suggestions.

According to the team leader, when discussing patients with compound issues, the patients’ situations were divided into appropriate sub-themes, which were each in turn linked to the corresponding professional’s perspective or role. This can be understood as an efficient distribution of tasks and responsibilities. However, although both the interview statement and the observation note emphasised the diversity of professional perspectives in the interprofessional teams, no instances of intersection between professions were observed. Instead, in these two examples, when different perspectives were advanced by workers from one field, those in the other fields neither directly grasped their suggestions nor contributed with further reflections. As an analytical reflection, when operating as parallel perspectives or as series of perspectives, the whole concept of interprofessional teamwork cannot be fully taken advantage of as the potential for generating new insights is reduced. Consequently, the lack of friction poses a risk to the potential function of diversity.

Relatedly, in interviews, several informants highlighted diversity as the base of interprofessional teamwork. One social worker described the interaction between diverse professionals with unique perspectives as a process of ‘synergy’:

The biggest advantage, I think, is that we all have different perspectives, and understand the patients differently. And when we put our minds together, work collectively, we manage to see the whole person and accomplish the provision of better services for the patients.

Similarly, a psychiatrist stated:

As both professionals and individuals, we are sensitive to different things. We are also blind to different things. There is quality in several professionals observing the patients and everyone having an equal opportunity for influence. The opposite gives no space for creative work.

In summary, this section highlights diversity as a natural characteristic of interprofessional teamwork. However, there is an important dynamic between diversity and the other two aspects. As both the social worker and the psychiatrists expressed, without friction, diversity risks not realising its full potential. Furthermore, the informants highlight a need for harmonisers such as ‘everyone having an equal opportunity for influence’, to connect diverse perspectives and to balance potential conflict. Correspondingly, both the last two excerpts suggest how the dynamic between diversity in perspectives (friction) promote creativity and synergy effects.

### Friction

Friction occurs when divergent perspectives and resources intersect during interactions between professionals, as exemplified in a team meeting discussion of a patient who primarily used his voice to scream and used little verbal language:

Discussing the reason behind this, one psychologist suggested: ‘The fact that [the patient] screams so much could indicate that [the patient] lacks the ability to express himself in words’.

A social educator who knew the patient well responded: ‘I think he screams to get his way’.

The phycologist countered: ‘But it could be something cognitive’.

A second social educator argued: ‘Yes, that could be, but I also think [the patient] screams to get his way’.

This discussion too ended with no clear conclusion. Unfortunately, the psychologist was never interviewed for this study, but both social educators were. When asked about the discussion, the first social educator reflected; ‘We cannot agree on everything. The most important thing is that different perspectives are raised.*’* As such, the social educator emphasised how consensus was not a goal of teamwork. Correspondingly, the second social educator said:

As a social educator, you tend to have a focus on behaviour. (…) What I hear when the psychologist asks, ‘could it be something cognitive?’ is a question of, ‘could it be a mental disorder?’ (…) It shows how we challenge each other. Had only social educators been discussing the case, maybe there would have been a complete consensus explaining it as behaviour.

Although the explanation proposed by the psychologist and the explanation proposed by the two social educators were not necessarily contradictory (either, neither, or both explanations could explain the patient’s screaming), the friction between the diverse professional perspectives is clearly illustrated in the interaction as different perspectives are present. Just as the social educators’ explanation can be traced back to the perspective of ‘behaviour analysis’, which is central in social education, the psychologist’s mental illness perspective reflects the focus of psychology training. Notably, both social educators emphasised how rather than posing a barrier to collaboration, intersections and discussion of different professional perspectives prevented professional bias and groupthink [28, 29] and created a potential ‘learning zone’ [30].

The following situation from a team meeting suggests that the intersection process promotes an ‘increase’ in a team’s shared knowledge compared to what might have been their starting point. Here, the team was discussing a patient they had been working with for a long time but whose progress had stalled. The discussion quickly turned to the underlying cause of the patient’s problems:

Social worker: ‘[Another social worker] and I had a meeting with [the patient] last week about his financial situation. He was very aggressive. We talked together after the meeting and decided that it may have been symptoms of withdrawal.’

Psychologist: ‘Intoxicated?’

Social worker: ‘Not intoxicated. He was aggressive and kept saying he needed medication. (…) What are your thoughts?’

Psychologist: ‘My notes say symptoms of anxiety and personality disorder. (…) I have not seen any signs of substance use, but you may have noticed something I have not.’

This situation demonstrates two things. First, due to differences in professional roles, the professionals met the patient in different contexts, and each can thus offer different insights into the patient’s life. In this case, the social workers met the patient in the context of the patient’s financial situation, and the psychologist meet the patient primarily in the context of therapy. Second, the social workers and the psychologist had different professional backgrounds for understanding the patient’s situation. While the psychologist’s knowledge base was mental health, the social workers’ training emphasised other factors, in this case, the patient’s living conditions. Consequently, when the two perspectives intersected, a new and deeper insight into the patient’s challenges was created. Although it is impossible to understand such ‘deeper insight’ as something measurable, it illustrates a situation where each team member's perspective collectively contributes to something ‘more’.

Even though many of the informants highlighted the function of friction positively, friction is not always straightforward. The data also points to the risk of friction becoming a barrier to collaboration, turning into conflict, or inhibiting some professionals from expressing their opinions, resulting in a form of professional self-sacrifice [24]. One psychologist, for example, felt that other team members were quicker to propose medication than what he was comfortable with but that he did not always assert his position. Reflecting on this, he said:

Personally, I think we should be restrictive in prescribing certain kinds of medication, but I do not always express that. (...) I do not bother taking on those discussions. Rather, I think, ‘okay, in addition to prescribing medication, can we also take other actions?’ (…) But this is a compromise according to what I think.

Not always wanting to express his opinion, this statement can be read as both a result of friction (the team members have different opinions) and a strategy to avoid friction (which may lead to conflict), and instead preserve harmonisation.

In the light of the previous discussion on diversity and parallel perspectives, it can seem like friction occurs when something is at stake. Disagreements on fundamental principles among team members can be problematic, as exemplified by the following excerpt from a nurse:

If there is a fundamental disagreement within the team concerning how we work, if these fundamental principles are constantly criticised, then that can be very destructive.

However, such conflict did not mean that all hope was lost. The same nurse stated:

Such disagreement has two sides. It can be destructive if the principles the team is founded on are constantly questioned. However, such discussions also make us aware of what we are doing and why.

The dynamic between friction and diversity and the two sides of its interaction can be clearly observed here. The main point, however, is that while competition between diverse perspectives can be motivating and effective, when not handled correctly (for example, if team harmonisation is missing), friction risks leading to conflicts, which can undermine the potential benefits of interprofessional work.

### Harmonisation

Harmonisation can bind distinct professionals and potentially opposing forces. Consistent with literature highlighting respect, trust, and the mutual acknowledgement of professional roles as conflict reducing factors [9–13], this study’s findings demonstrate how such harmonising elements function to connect team members. For example, when asked how the team members worked together despite professional differences, several informants emphasised the ability to learn and being responsive to the perspectives of other professions as an important factor in being a ‘trustworthy team member’. A psychologist, who had joined the team quite recently expressed in an interview: ‘I have spent a lot of time learning to understand the other [professionals] in the team and how they work.’ Likewise, one of the nurses stated:

You must be interested in the perspectives of others and be willing to learn and contribute to the discussions. If not, you should not be part of this team.

In addition to the ability to learn and being responsive to the perspectives of other professionals, several informants expressed that they worked well together when all opinions and perspectives were equally valued: A social worker told the researcher in an interview:

In the beginning, I dared to say almost nothing. It took some time, but when I realised that the others listened to me and that my perspectives were appreciated, well, that was very reinforcing and gave me confidence. (…) When you feel that you are listened to and that your opinion is valued, you also contribute well as a team member.

The social worker connected feelings of being respected and valued with ‘contributing well as a team member’. This statement was consistent with other informants’ emphasis on the importance of ‘all voices counting equally’. A psychiatrist observed the following:

Everyone [on the team] is allowed to voice their opinion. Even if one of the ‘heavyweights’, me for example, expresses something, there must be room for others to say, ‘you know what? I do not agree. I see it differently’. (…) We have succeeded in this because [the team members] have confidence in each other.

Here, the psychiatrist linked the space for utterance to the team members having confidence in each other. Such expression of egalitarianism can be seen as a strategy to downplay hierarchical differences and preserve harmonisation within the team. Simultaneously, it is of interest how the psychiatrist expresses awareness of his higher status compared to other team members by characterising himself as one of the ‘heavyweights’, and thus highlighting how it can be difficult for professionals with lower status to contribute with their perspectives [18, 19]. Unfortunately, the data does not contain any similar statements from ‘lower status’ professions confirming or disconfirming the psychiatrist's statement. However, when questioned about ‘what brought the team together’, a psychologist answered:

My perception is that we are a flexible group of professionals dedicated to helping the patients. (…) We are collectively responsible for finding solutions that correspond to the patients’ needs, and that is what unites us.

In this statement, the psychologist expressed how shared responsibility and a shared goal (responding to the patients’ needs) created a ‘bond of commitment’ between the team members.

The above interview excerpts shed light on some important functions of harmonisation. Yet, locating similar expressions during ‘real-time interaction’, that is, during team meetings, was comparatively difficult. One reason for this may be that there is a discrepancy between what people say and what they ‘do’, meaning that harmonisation was something the informants expressed through words rather than actions. Another reason may be that such interpersonal elements are subtle and difficult to observe in actual practice. The team meetings, were, however, often used as forums for professionals seeking support for their approaches towards working with patients. In those situations, team members were often observed making brief affirmative comments to each other, such as ‘I agree’, and ‘well spotted’, or nodding their heads affirmatively. Such affirmations appear to be important expressions of support, helping to establish mutual trust, respect, and recognition among the professionals.

These findings suggest that relations based on mutual respect and trust, openness, and acknowledgement of each other’s professional roles can function as conflict reducing and promote a balanced composition of individual parts. However, as previously discussed, over-harmonising runs the risk of discouraging disagreement and debate between team members, which may lead to poor decision making and groupthink [28]. Harmonisation must not stand alone. Reflecting on the dynamic between diversity, friction, and harmonisation, one team leader said:

That [the team members] involve themselves and share their different views is in many ways praiseworthy. Professional friction creates space for professional growth. However, it is also a source of conflict and a challenge if they have very diverse perspective on an issue. (…) It is an interesting but difficult question. One important factor, I think, is a sense of confidence within the team, that we know that disagreement is okay and that the team can survive a conflict.

Here, the team leader emphasises how all three aspects, seen collectively, support an effective interprofessional teamwork dynamic. Especially the team leader highlights friction and the need for harmonising elements such as trust as important factors. However, the empirical data also gives the impression of a dominant need to over-promote harmonisation and its positive function. For example, several of the informant's statements seemed to under-communicate differences in professional status and, instead, emphasise how ‘all voices counted equally’. Exactly why this was so, is difficult to answer. One suggestion may be that diversity and friction are, to some extent, perceived as barriers to effective teamwork [16], while the balancing benefits of harmonisation are described as important to accomplish team goals [11]. Such an excessive focus on harmonisation can lead to the disappearance of the potentially positive functions of diversity and friction.

### The threefold model

Based on the hypothesis that diversity, friction, and harmonisation have important functions in interprofessional teamwork and can also involve certain risks, this article examines how, when combined, those aspects may create conditions conducive to effective teamwork. Findings from analysis of the data indicate that the three aspects are closely linked, as illustrated in the table below (Table 3):

**Table 3:** Threefold model of interprofessional team dynamics

	Function	Risk	Dynamic
**Diversity**	- Expanding access to knowledge and different perspectives - Distributing tasks and responsibility	- Parallel rather than intersecting perspectives	- Promotes friction and calls for harmonisation.
**Friction**	- Advancing innovation and broader, deeper discussions - Generating new insights	- Conflict	- Counteracts risks for parallel perspectives, the risk for groupthink and over-harmonisation.
**Harmonisation**	- Supporting a balance of professional distinctiveness - Tying team members together.	- Groupthink	- Reduces risks of conflict - Creates a ‘psychological space for friction’ where opposing perspectives are encouraged and accepted.

New characteristics of these aspects in the context of interprofessional team dynamics have not been discovered here; numerous studies have contributed to knowledge on interprofessionalism and teamwork relations, exploring the effects and characteristics of each aspect in practice [9, 10]. What is new, is an emphasis on the dynamic and balance between them, which allows for a more comprehensive discussion and understanding of processes that promotes effective teamwork. For example, compared to previous studies which have highlighted diversity in professional cultures and roles as a challenge to effective interprofessional teamwork [16, 25], this study points to diversity as the very basis of interprofessional teamwork and that for diversity to reach its full potential, friction must occur. Here, the findings suggest that friction promotes innovation by producing new insights and taking discussions further. Lastly, harmonisation was found to support a balance in professional distinctiveness, as it fosters trust, respect, and ties professionals together.

The dynamic between the three aspects indicates important processes in interprofessional teamwork. This was, for example, observed in team meeting discussions where the intersection of different professional perspectives helped the team members expand understanding of a patient's situation. For the sake of clarity, the three aspects have been presented separately. However, what is important is how they work together in practice.

By considering the three aspects in combination, an ideal-type model is presented that emphasises the practice of interprofessional teamwork and the risk if any of the aspects are missing or becomes to dominant. Here, the data suggests that the team members seemed to seek a balance between the three aspects - which they sometimes succeed with, other times not. However, the finding also indicates that if professionals on a team lack mutual trust or respect (harmonisation), differences between them and resulting frictions can lead to conflict. Conversely, the lack of diversity, disagreement, and debate between team members can result in collective rationalisation, self-censorship, and poor decision-making [28, 29].

### Limitations and suggestions for further research

Though the model presents a simple design, it was useful for examining three important aspects of interprofessional teamwork, as expressed in both the professionals’ stated motivations and their observed actions in practice. However, as stated earlier, this should only be understood as an ‘ideal-typical model’ of interprofessional team dynamics [5], meaning that it presents the pure features of a phenomenon cleansed of anything that might oppose it. For example, although power structures are clearly present in interprofessional teams, they are not treated at length in this article (but in a forthcoming one, based on the same study). By considering differences in professional status among team members, future studies may offer a deeper understanding of the three aspects and how they are connected. It would also be interesting if further research explored how the dynamics between the three aspects inform team actions. A further limitation is that, when discussing effectiveness, no objective assessment of results and achievement of goals was included. Instead, an evaluation was made based on the informants’ subjective perceptions. This means that the study cannot say anything about what effective teamwork actually is. However, it presents an empirically grounded ideal-type model.

In the field of mental health and substance use, there is a great deal of overlap between the professions involved. Interprofessional teams made up of multiple professionals from different fields with the right to voice their opinions to one another, may enable greater levels of friction than other professional environments. It could be of interest to explore the operations of the three aspects within different contexts where there are greater differences between the professionals on the team and a greater potential for conflict. It would also be of interest to investigate how cultural norms affect teamwork dynamics. In Norway, for example, the work culture is characterised by informal communication, a high degree of autonomy for workers, and a high level of trust [38]. This may have affected how the professionals in this study acted and expressed themselves. In other societies it may look different. Lastly, what this study does not account for is how teamwork develops over time. For example, it is possible to imagine that disruptive friction is most at risk when starting up a team, at the same time as elements of stabilisation and harmonisation take time to develop.

## Conclusion

By analysing the three aspects in combination, this study suggests that connections between diversity, friction, and harmonisation can directly impact interprofessional teamwork outcomes. This ideal-typical model and the findings presented contribute to research on interprofessional teams through its comprehensive, analytical presentation of interprofessional team dynamics. By considering the three aspects in combination, the model suggests risks to interprofessional teamwork if an aspect is missing or comes out of balance. This study also contributes to the field of health services practice and development by offering professionals an exploratory model for examining their own team dynamics.

## Data Availability

The datasets generated and analysed during the current study are not publicly available to assure the informants’ anonymity. The corresponding author can be contacted for questions about the data.
